# Anthropometrics, Hemoglobin Status and Dietary Micronutrient Intake among Tanzanian and Mozambican Pigeon Pea Farmers

**DOI:** 10.3390/nu14142914

**Published:** 2022-07-15

**Authors:** Laila Eleraky, Ramula Issa, Sónia Maciel, Hadijah Mbwana, Constance Rybak, Jan Frank, Wolfgang Stuetz

**Affiliations:** 1 Institute of Nutritional Sciences, University of Hohenheim, 70599 Stuttgart, Germany; laila.eleraky@nutres.de (L.E.); ramulajuma.issa@uni-hohenheim.de (R.I.); jan.frank@nutres.de (J.F.); 2 Faculty of Health Sciences, Lúrio University, Nampula 3100, Mozambique; smaciel@unilurio.ac.mz; 3 Department of Human Nutrition and Consumer Sciences, Sokoine University of Agriculture, Morogoro 3006, Tanzania; hadija27@yahoo.com; 4 Leibniz Centre for Agricultural Landscape Research (ZALF), 15374 Müncheberg, Germany; constance.rybak@zalf.de

**Keywords:** legumes, dark green leafy vegetables, Tanzania, Mozambique, anemia, micronutrient intake, micronutrient status, overweight, small-scale farmers

## Abstract

Inadequate consumption of micronutrient-dense and protein-rich foods such as vegetables, legumes and meat is an important contributing cause for anemia and deficiencies of vitamin A and iron in rural communities of Tanzania and Mozambique. A cross-sectional study was conducted to assess the nutritional status (anthropometrics and hemoglobin) and diets in particular micronutrient intake of female and male pigeon pea farmers from Lindi, Tanzania, and Gurué, the Zambézia province of Mozambique. A total of 1526 farmers (669 from Tanzania, 857 from Mozambique) were studied, of whom 16% were overweight and 35% were anemic. The highest prevalence of overweight and anemia, at 35% and 48%, was observed in Tanzanian and Mozambican women, respectively. Overall, only a small proportion of women and men reached the recommended daily dietary intake of vitamin A (10%), iron (51%) and zinc (44%). Multiple regression models revealed that dark green leafy vegetables (DGLVs) highly predicted vitamin A intake, whereas legumes in Tanzania and starchy plants in Mozambique were actually the dominant sources of vitamin A. Cereals covered over half of the iron and the zinc intake in both countries. An increased consumption of micronutrient-rich DGLVs and legumes, while reducing the high amounts of refined maize or polished rice, is suggested to counteract the high prevalence of anemia and overweight among smallholder farmers in East and South Eastern Africa.

## 1. Introduction

Anemia is a worldwide problem that is highly prevalent in developing countries, especially in women of reproductive age [1]. Infectious diseases, poor water hygiene, and the low bioavailability of dietary iron from specific (plant-based) foods are the main contributing factors for a high prevalence of anemia [2]. Rural areas of Sub-Saharan Africa are among the most affected regions with anemia and deficiencies of vitamin A, iron and zinc [3,4,5]. Malaria infection was positively associated with having anemia according to a study on women of reproductive age (15–49 years) enrolled in the demographic and health surveys of 27 countries in Sub-Saharan Africa, conducted between 2010 and 2019 [6]. Blood loss caused by worm infections including hookworm, whipworm and schistosomiasis leads to direct iron-deficiency anemia [7]. A previous case–control study among 191 anemic and 382 non-anemic pregnant women from Ethiopia revealed anemia as almost 6-fold more common among pregnant women who had intestinal helminthic infection than those with no intestinal helminthic infection [8]. In the Lindi district of Tanzania, nutritional anemia and iron deficiency were associated with a monotonous cereal and vegetable-based diet, as well as parasitic and malaria infection [9]. A cross-sectional study of more than 500 female adolescents from rural and urban Mozambique examining their biochemical status showed that the prevalence of deficiencies and dietary intake revealed almost every second female as anemic (42.2%) on account of iron depletion and have low serum zinc and vitamin A deficiency due to diets rich in carbohydrates and low in protein, fat and micronutrients [10].

A general trend of nutrition transition in low-income countries regarding a transformation in the diets towards less healthy ultra-processed foods rich in refined carbohydrates, fat and sugar led to the emergence of ‘a double burden of malnutrition’ by means of both undernutrition and overweight [11]. In the case of an additional micronutrient deficiency, one defines a ‘triple burden of malnutrition’, namely undernutrition, overweight and micronutrient deficiency in the same population [12]. The persistent dietary deficiencies of particularly vitamin A, iron and zinc alongside emerging overweight occurring within the same community have already been proven many times, as illustrated in a study of adolescents from four rural and urban settings in Africa and India [13]. In the latter study, both undernutrition and overweight were present in all settings and adolescents reported low intakes of micronutrient-rich fruits and vegetables, but high intakes of sweets and sweetened soft drinks. The prevalence of overweight and obesity increased significantly between the years 1991 and 2014 according to demographic and health survey data of women 15–49 years old from 24 African countries, including Tanzania and Mozambique [14]. A survey from Sub-Saharan Africa including 276 adults from Tanzania revealed three in four participants from Tanzania as overweight or obese and women as more affected by overweight than men [15]. The double burden of malnutrition characterized by the coexistence of undernutrition along with overweight and even obesity has emerged as an important health problem in Sub-Saharan Africa and is directly linked to the increase in ultra-processed food consumption [16].

A higher consumption of micronutrient-rich dark green leafy vegetables (DGLVs) and protein-rich legumes such as pigeon peas (*Cajanus cajan*) would positively contribute to a healthy diet, as shown in Botswana [17]. Pigeon peas are an excellent source of proteins, and also provide high concentrations of minerals including iron and zinc [18,19,20]. A higher consumption of DGLVs was associated with higher hemoglobin and a lower prevalence of vitamin A and iron deficiencies in resource-poor communities, as shown in a previous study on 666 women from different areas of rural Tanzania [21]. The Vegi-Leg project, a multidisciplinary study supported by the German Federal Ministry of Food and Agriculture (BMEL), operates in Lindi, South Tanzania, and in Gurué, the Zambézia province of Mozambique, among female and male pigeon pea farmers. The project aims to improve the nutritional status of both female and male farmers by introducing tailored, nutrition-sensitive interventions including improved processing technologies, e.g., suitable drying techniques for pigeon peas and dark green leafy vegetables to ensure year-round food security. The objective of the baseline survey held in 2019 was to assess nutritional status by measuring anthropometry and hemoglobin and to identify nutritional gaps, by calculating dietary micronutrient intake among the enrolled female and male farmers in the study regions.

## 2. Materials and Methods

### 2.1. Study Population and Field Procedure

The baseline study of the Vegi-Leg project was carried out in July to August 2019 in the Mitumbati and Mibure villages in Lindi, Southern Tanzania and in the Nicoropale and Ruace villages of Gurué district, in the Zambézia province, Central Mozambique. Inclusion criteria for the Vegi-Leg project was that the farmers had grown pigeon pea in the last 3 years. The study population were female and male small-scale pigeon pea farmers in regions where a high prevalence of anemia (women from Lindi in Tanzania 48.9% and from Zambézia in Mozambique 61.7%) was previously reported [22,23,24].

The study procedure included anthropometric and hemoglobin measurements, as well as 24 h dietary food intake recalls of the farmers.

The surveys were carried out according to the guidelines laid down in the ‘Declaration of Helsinki’ and approved by the National Institute for Medical Research, Dar es Salaam and the Ministry of Health, Community Development, Gender, Elderly and Children in Dodoma, Tanzania (NIMR/HQ/R.8a/Vol.IX/3040), and ethically reviewed by the National Bioethics Committee for Health of Mozambique (IRB00002657, Ref 370/CNBS/19). Written informed consent was obtained from all farmers. 

### 2.2. Anthropometric and Hemoglobin Measurements

Weight, height and mid-upper arm circumference (MUAC) were measured using electronic or mechanical floor scales (Seca 874 and Seca 750, Seca GmbH & Co KG, Hamburg, Germany), a wooden (UNICEF) or plastic stadiometer (Seca 213, Seca GmbH & Co KG, Hamburg, Germany), and a standard MUAC tape (UNICEF in Tanzania or Seca 201 in Mozambique), respectively. Weight was recorded to the nearest 0.1 kg, while height and MUAC were measured to the nearest 0.1 cm. MUAC was measured on the left arm. Body mass index (BMI) was calculated from weight and height measured at admission; cut-offs for underweight (<18.5 kg/m^2^), overweight (≥25 kg/m^2^) and obesity (≥30 kg/m^2^) were used according to WHO and FANTA instructions [25,26]. MUAC cut-offs demonstrating underweight at <24 cm, overweight at ≥30 cm and obesity at ≥33.5 cm were set based on suggestions from recent studies [27,28].

Hemoglobin concentrations were measured at the study site using capillary blood sample obtained from a finger prick by using a sterile lancet measured into a hemoglobinometer (HemoCue Hb 201+, HemoCue AB, Ängelholm, Sweden). For quality control, a “HemoTrol” quality control solution (normal, 12 g/dl, Eurotrol, Elizabethtown, KY, USA) was measured daily for the 23 days (5–8 times per day), giving an overall mean (SD) of 12.2 (0.32) g/dl and a coefficient of variation (CV) of 2.6%. Anemia was defined as hemoglobin <120 g/L for non-pregnant, <110 g/L for pregnant women and <130 g/L for men [29].

### 2.3. Assessment of Dietary Intake

Information on the amount of consumed food items was collected using 24 h food recalls. For the 24 h recalls, each interviewee was asked to precisely name all foods and amounts consumed in the past 24 h. Photos of portion sizes using typical household utensils in the form of booklets were prepared and used to estimate the amounts consumed and subjects were asked to indicate if the named amount was consumed alone or shared in the household. The macro- and micronutrient intake of the female and male farmers was calculated using the Tanzanian Food Composition Tables [30] and a Mozambican food database from a former study in the same region, the Zambézia province [31], respectively. Macro- and micronutrient food values of those food databases were imported into NutriSurvey software (www.nutrisurvey.de) (accessed on 29 February 2020) [32]. The NutriSurvey database was completed with a Kenyan food database, as well as micronutrient concentrations of DGLVs previously analyzed at the university of Hohenheim [33].

The calculated intakes of macro and micronutrients were compared with daily recommended dietary intakes according to sex, age and pregnancy status [34,35]. Individually assessed food items (e.g., grams of eaten mango or cassava) were further merged to 14 food groups to assess their frequency and amount consumed and to finally elucidate different food groups responsible for iron, vitamin A, and zinc intake.

The 14 food groups are defined as follows: 1. ‘Cereals’: maize, rice, sorghum, and millet (‘ugali’ and ‘uji’); 2. ‘Bread and similar products’: dough made from wheat or rice fried or baked in oil (bread, chapatti, noodles, African donuts, ‘bhajia’, and half-cake); 3. ‘Starchy plants’: white potatoes, sweet and Irish potatoes, cassava, plantain, yam, and taro; 4. ‘Legumes’: pigeon peas, cowpeas, kidney beans, lentils, chickpeas, and bambara nuts; 5. ‘DGLVs’: cowpea leaves, spinach, amaranth leaves, cassava leaves, sweet potato leaves, mlenda, lettuce, and pumpkin leaves; 6. ‘Other vegetables’: tomatoes, eggplant, carrots, onions, okra, cabbage, pumpkin, and mushroom; 7. ‘Fruits’: mango, papaya, banana, jackfruit, avocado, orange, lemon, and mandarin; 8. ‘Nuts’: groundnuts, cashews, coconut, and sesame; 9. ‘Oil and fat’: sunflower oil, vegetable oil, palm oil, and butter; 10. ‘Fish’: fresh or dried fish, sardines, fried fish, tuna, and shrimp; 11. ‘Meat’: beef, chicken, duck, rat, goat, and pork; 12. ‘ Eggs and diary’: egg pancakes, eggs, milk, and yoghurt; 13. ‘Sugar and sweets’: sugar, sugarcane, carbonated drinks, cola, and sweetened juices; 14. ‘Beverages’: tea and beer.

### 2.4. Statistical Analysis

Demographic characteristics, anthropometrics, hemoglobin, and the calculated macro- and micronutrients intake of the farmers were described using medians and interquartiles (25% and 75% percentiles) for continuous variables and percentages for categorical data. Socio-demographic data, micronutrient intake and frequency and amount of consumed food groups were compared between female and male farmers of the two different study regions in Tanzania and Mozambique using the Kruskal–Wallis test with pairwise multiple comparisons or the Chi-Square test, as appropriate.

For further analysis regarding multiple regression, dietary intake data on vitamin A, iron and zinc were transformed using square root (SR) or logarithmic (LN) transformation to achieve normal distribution. For each of the two study sites, multiple linear regression models with a forward stepwise approach were applied to identify predicting food groups (e.g., grams of leafy vegetables or legumes) for vitamin A, iron and zinc intake. In addition, the percentage contribution of the respective food groups to vitamin A, iron and zinc intake was calculated as the product of frequency and average consumption of the food groups.

All statistical analyses were conducted using SPSS Version 20; *p* values < 0.05 were considered as statistically significant.

## 3. Results

A total of 1526 farmers—669 from Lindi, Tanzania and 857 from Gurué, Mozambique—were successfully enrolled in the Vegi-Leg baseline study. Socio-demographic data, anthropometrics and hemoglobin concentrations of the female (55.7%) and male (44.3%) farmers, separated by region (Lindi) or district (Gurué) of the respective countries, are summarized in Table 1. Female and male farmers from Tanzania were significantly older (+7 years for females and +5 years for males) than those from Mozambique.

Weight and height were higher among male compared to female farmers in both countries, but BMI, MUAC, and the prevalence of overweight and obesity were significantly higher in women (in both countries) than in men. In Tanzania, one in three women was overweight and one in ten was obese. Underweight only played a role among men in both countries, as the prevalence of underweight was higher than the prevalence of overweight. In total, 35% of the farmers studied were anemic. The prevalence of anemia was significantly higher in women than in men and overall, significantly higher in Mozambique than in Tanzania: each second woman in Mozambique and each third woman in Tanzania was anemic. Self-reported malaria was particularly high in Mozambique: one in two women and one in three men reported having malaria within the past 90 days; whereas one in six female or male farmers in Tanzania reported having malaria within the past three months.

The farmers’ reported dietary intake of energy, protein, fat, carbohydrates, vitamins and minerals of the previous 24 h in relation to the D-A-C-H [34] and FAO/WHO [35] recommended daily intakes (RDI), by country and sex are shown in Table 2. The reported food intake of male farmers from Tanzania revealed the highest energy, protein, fat and carbohydrates content and was significantly higher than that of female farmers in Tanzania and the farmers in Mozambique. Overall, farmers from Tanzania had a better protein intake; three out of four farmers in Tanzania achieved the RDI (75%), whereas only one out of two farmers achieved the RDI (50%) in Mozambique. Generally, the farmers in Tanzania were better supplied with vitamin A than the farmers in Mozambique; however, only the minority of farmers (5% in male farmers in Mozambique to 14% in male farmers in Tanzania) reached the daily intake recommendations for vitamin A. Even fewer farmers from both countries reached the recommended values for daily vitamin E intake (5–8%).

The farmers from Tanzania were better supplied than the farmers from Mozambique with regard to vitamin B2, folic acid and vitamin B12, whereas there was barely any difference between the countries for vitamin B1 and B6, with the lowest intake in women from Tanzania. In contrast, the intake of vitamin C was double as high in farmers from Mozambique than farmers from Tanzania. Farmers from Tanzania showed a significantly higher intake of calcium in comparison to farmers from Mozambique; however, only a small proportion of Mozambican (7–10%) and Tanzanian farmers (15–16%) reached the recommended daily intake of calcium. In contrast, farmers in both countries showed an adequate intake of magnesium, with approximately 80% of the RDI. Women had a similar intake of iron as men, whereas only one in three women reached the gender-specific RDI for iron, compared to two in three men. The zinc intakes were lower in Mozambique than in Tanzania, with the lowest intake among Mozambican men.

Table 3 summarizes the frequency and the median quantity of consumed foods according to the 14 food groups, as reported in the 24 h recalls. The portions consumed were determined using a booklet with pictures of different defined (e.g., 125 g, 250 g or 500 g) portion sizes of food weighed in advance to allow a more accurate assessment by the farmers. In Tanzania, almost all female and male participants (98%) reported the consumption of ‘cereals’, while in Mozambique 82% of female and 76% of male farmers had cereals the previous day. In terms of quantity, men from Tanzania have recorded the highest consumption of cereals. Legumes were the second most consumed food group in both countries, with both frequency and quantity consumed significantly higher in Tanzania than in Mozambique. ‘Starchy plants’ were more frequently and in larger quantities consumed in Mozambique compared to Tanzania. Further, female and male farmers from Mozambique consumed ‘fish’, ‘meat’, ‘other vegetables’, ‘sugar and sweets’ more often, but the Tanzanian farmers consumed these foods in greater quantities. Significantly more farmers from Tanzania than from Mozambique reported the consumption of energy-dense carbohydrates from ‘bread and similar products’, ‘fruits’ and ‘beverages’, which include tea, coffee and alcohol, whereas the consumption of ‘green leafy vegetables’ in Mozambique was significantly higher than in Tanzania.

The food groups consumed (in grams) as predictors and their quantitative contribution to the intake of vitamin A, iron and zinc intake are presented in Table 4. For vitamin A, DGLVs was the most significant predictor of vitamin A intake in both countries (partial R), while legumes in Tanzania and starchy plants in Mozambique were quantitatively responsible for 50% and 42% of vitamin A intake, respectively.

Legumes and cereals, in Tanzania, and cereals, in Mozambique, were the main predictors of iron intake; and legumes, cereals and fish, in Tanzania, while again cereals, in Mozambique, were the most relevant predictors of zinc intake. In Tanzania, cereals followed by legumes were also the most important food groups for iron and zinc intake in terms of quantity, while in Mozambique cereals followed by starchy plants and legumes were the quantitatively predominant food groups for both iron and zinc intake.

## 4. Discussion

The present study confirms the trend of an increasing prevalence of overweight and obesity, joint with a simultaneously high prevalence of anemia and deficient micronutrient intake in East-African female and male farmers [36,37].

Significant differences between farmers from Lindi, Tanzania and Gurué, Mozambique in terms of frequently and quantitatively consumed food items, micronutrient intake and respective nutritional status were evident. Women of both countries had a significantly higher prevalence of overweight and obesity compared to men, but at the same time a lower dietary intake of iron, significantly lower hemoglobin concentrations and accordingly a higher prevalence of anemia. Hence, it was observed that men were only half as often affected by anemia compared to women from both countries who were twice as likely to have reached the recommended daily intake (RDI) for iron intake. Low iron intake in combination with low or limited iron bioavailability, caused by a high concentration of the anti-nutrients phytate and tannin present in, e.g., black tea and plant-based diets and particularly in beans, could be one explanation for the higher prevalence of anemia in female farmers of our study [38,39]. The insufficient dietary intake of vitamin A and pro-vitamin A active carotenoid in female and male farmers of the present study, where 9 in 10 farmers did not reach the daily intake recommendations may have further reduced the iron bioavailability. Vegetables rich in β-carotene are able to significantly improve the bioavailability of iron from cereals and pulses as verified in a study on corn, wheat and rice, explaining that β-carotene forms a complex with iron, keeping it soluble in the intestinal lumen and preventing the inhibitory effect of phytate on iron absorption [40]. Ascorbic acid from fruits, which were rarely consumed in both countries can similarly enhance the absorption of iron from vegetarian diets [41]. Previous studies reported that traditional food preparation and cooking methods (fermentation, boiling, or frying) with reduced cooking time can significantly reduce phytate content in vegetables [42,43]. Various analysis of DGLVs confirmed them as excellent sources of β-carotene, iron and other minerals [44,45]. Nevertheless, according to the dietary assessment of the farmers of this study only every fifth person in Tanzania and every second person in Mozambique reported consuming DGLVs. A study among 90 pregnant women from Tanzania showed significant improvements of vitamin A status (plasma retinol) through an increased consumption of green leafy vegetables with oil, which increases the bioavailability of pro-vitamin A carotenoid [46]. Another study on 666 mother–children pairs from two different districts of rural Tanzania proved that the consumption of whole-grain millet with DGLVs led to improved blood iron status (mothers and children) and better vitamin A status (retinol in serum of mothers) compared to processed grains with legumes, which consequently resulted in a significantly lower prevalence of anemia and iron deficiency [21,47]. In the present study, the prevalence of anemia and the deficient dietary intake of vitamin A and iron was the highest in female farmers from Mozambique. Every second woman (48%) was anemic and every second woman (53%) also reported having malaria in the last three months. The identified effect of malaria infections on erythropoiesis and the iron recycling that occurs after parasite-induced hemolysis may be responsible for the high anemia rates [48], as previously shown in African refugees [49]. According to the 24 h recalls of the female and male farmers of both countries, their main meal consisted of cereals in form of polished rice or maize combined with a serving of legumes. Large amounts of refined carbohydrates and too few micronutrient-rich vegetables (DGLVs) and food of animal origin are most likely the cause for the existing multiple burden of overweight/obesity, anemia, and insufficient dietary intake of vitamin A, iron and zinc, as also shown in previous studies [50]. These inadequate and insufficiently diversified eating habits have been already reported in Tanzanian farmers from Morogoro, with diets consisting of less than six food groups of a total of 12 defined possible food groups, and unmet dietary micronutrient intake recommendations [51]. A high intake of refined grains was recently associated with a higher risk of obesity, major cardiovascular disease events and mortality [52]. The general nutrition transition characterized by the paradoxical coexistence of micronutrient deficiencies, underweight and pre-obesity/obesity has been already noted frequently in urban and rural Africa, as obesity becomes a condition more associated with poverty due to the increasing affordability of highly refined oils, sugar-sweetened beverages and carbohydrates [53,54]. In the present study, the significantly more frequent consumption of sugar-sweetened beverages in Tanzania compared to Mozambique (57–63% vs. 6–7%) is very likely one of the causes for the higher prevalence of overweight there. A review from 2014 also demonstrated how the increase in sugar intake, with an intake higher than the recommended 40 g per day, was a powerful predictor of overweight and obesity in countries of Sub-Saharan Africa, including Tanzania [55,56]. A recent study, conducting data between 2008 and 2017, from 23 countries of Sub-Saharan Africa including Tanzania and Mozambique found out that a triple burden of malnutrition (anemia, underweight and overweight) was present in 5–8% of the households and that interventions must address overweight/obesity but also undernutrition and anemia [57]. A study on 976 adolescents from Ghana stated an even higher prevalence of overweight than in the present study with almost half of female and male adolescents (48%) overweight, while approximately one-third of males (30.5%) and half of females (52.2%) had mild (females:110–119 g/L, males: 110–129 g/L) to severe (<80 g/L) anemia [58,59]. According to the WHO classification of public health significance, the prevalence of anemia was ≥40%, as detected in the female farmers in Mozambique of the present study, and the female adolescents of the study from Ghana is considered a severe global public health problem [59]. A similarly high prevalence of overweight (53%) and anemia (38%) was reported in a national study among non-pregnant women (n = 3089, 15–49 years) from Azerbaijan, where the rates of vitamin A deficiency (11%), iron deficiency (34%) and iron-deficiency anemia (24%) were substantially high [60].

In addition to the power of the present study, there were limitations such as the cross-sectional design and thus single time point of hemoglobin measurement and dietary intake. Further, common under-reporting of portion sizes and of consumed sugar in teas or porridges in the 24 h recalls might have biased the food intake in terms of energy of the farmers in this study. Another limitation is that we measured deficiencies of micronutrients through dietary intake only; further, hemoglobin was measured, but unfortunately, we did not take blood samples for the measurement of biological markers; here, blood parameters such as ferritin and haptoglobin would have been beneficial to determine the status of iron deficiency in relation to measured anemia rates. The strengths of this study include the large sample size and the assessment of both female and male farmers from two different villages in each of the regions studied, Lindi in Southern Tanzania and Zambézia in North-western Mozambique. All enrolled farmers were measured (anthropometry and hemoglobin) and interviewed by two well-trained teams within 6 weeks.

## 5. Conclusions

In conclusion, the outcomes of the present study suggest a strong impact of dietary habits on the prevalence of overweight and micronutrient deficiencies among Tanzanian and Mozambican female and male small-scale pigeon pea farmers.

In terms of overweight, anemia and deficiencies of vitamin A and iron, a micronutrient-rich diet with whole grains, more legumes and especially DGLVs would be advantageous over a diet consisting mainly of polished maize or rice and insufficient amounts of vegetables, legumes and fruits. Further, interventions within the Vegi-Leg project, including nutritional educational programs and the development and introduction of proper drying techniques for leafy vegetables and pigeon peas that conserve micronutrients, are promising measures to reduce the high prevalence of anemia, micronutrient deficiencies and overweight among small-scale farmers of the study areas.

## Figures and Tables

**Table 1:** Characteristics and anthropometrics of the study population.

	All (N = 1526)	TZ-F (N = 362)	TZ-M (N = 307)	MZ-F (N = 488)	MZ-M (N = 369)
Age [years] ^1^	39.0 (28.0, 52.0)	41.0 ^a^ (31.0, 57.0)	44.0 ^a^ (32.0, 58.7)	34.0 ^b^ (25.0, 45.0)	39.0 ^c^ (27.0, 51.0)
Weight [kg] ^1^	52.7 (47.9, 59.2)	53.8 ^a^ (47.9, 62.3)	55.0 ^a^ (49.3, 61.2)	50.5 ^b^ (46.0, 56.5)	53.0 ^c^ (49.9, 59.0)
Height [cm] ^1^	156.1 (151.1, 162.1)	151.8 ^a^ (148.3, 155.4)	162.1 ^b^ (158.0, 166.7)	153.1 ^c^ (149.0, 156.8)	162.0 ^b^ (157.2, 166.7)
BMI [kg/m^2^] ^1^	21.3 (19.6, 23.7)	23.5 ^a^ (21.0, 26.4)	21.0 ^b^ (19.5, 22.7)	21.5 ^c^ (19.9, 23.7)	20.2 ^d^ (19.1, 21.7)
<18.5 kg/m^2^	12.0 (183)	6.9 (25)	13.6 (42)	10.9 (53)	17.1 (63)
18.5–24.9 kg/m^2^	72.0 (1098)	58.0 (210)	78.2 (241)	73.6 (359)	78.3 (288)
25–29.9 kg/m^2^	12.3 (187)	23.8 (86)	7.1 (22)	13.5 (66)	3.5 (13)
≥30 kg/m^2^	3.8 (58)	11.3 (41) ^a^	1.0 (3) ^b^	2.0 (10) ^c^	1.1 (4) ^b^
≥25 kg/m^2^	16.0 (245)	35.1 (127) ^a^	8.1 (25) ^b^	15.6 (76) ^c^	4.6 (17) ^d^
MUAC [cm] ^1^	27.1 (25.4, 29.1)	28.1 ^a^ (26.1, 31.5)	26.8 ^b^ (24.7, 28.5)	27.3 ^c^ (25.6, 29.5)	26.4 ^b^ (24.9, 28.0)
MUAC < 24 cm, % (n) ^2^	10.1 (154)	7.2 (26) ^a^	14.9 (46) ^b^	6.4 (31) ^a^	13.8 (51) ^b^
MUAC ≥ 30 cm, % (n) ^2^	14.6 (223)	22.4 (81) ^a^	8.8 (27) ^b^	18.0 (88) ^c^	7.3 (27) ^b^
MUAC ≥ 33.5 cm, % (n) ^2^	4.5 (69)	11.0 (40) ^a^	1.3 (4) ^b^	4.3 (21) ^c^	1.1 (4) ^b^
Hemoglobin, g/L ^1^	129 (117, 140)	127 ^a^ (117, 136)	140 ^b^ (129, 152)	120 ^c^ (109, 129)	138 ^d^ (126, 148)
Anemia, % (n) ^2^	34.6 (528)	29.0 (105) ^a^	25.0 (77) ^b^	48.0 (234) ^c^	30.4 (112) ^a^
Pregnant, % (n) ^2^	3.6 (55)	3.0 (11)	-	9.0 (44)	-
Diarrhea, 4 weeks, % (n) ^2^	2.4 (37)	4.1 (15) ^a^	1.6 (5) ^b^	2.9 (14) ^c^	0.8 (3) ^b^
Malaria, 90 days, % (n) ^2^	33.8 (516)	17.7 (64) ^a^	16.3 (50) ^a^	53.5 (261) ^b^	38.2 (141) ^c^

TZ = Tanzania, MZ = Mozambique; TZ-F = TZ-female, TZ-M = TZ-male, MZ-F = MZ-female, MZ-M = MZ-male, BMI = body mass Index, and MUAC = mid-upper arm circumference. Data are the median (interquartile range) ^1^ and percentage (number) ^2^. Differences between individual groups were assessed by the Kruskal–Wallis and pairwise multiple comparison tests or the Chi Square test as appropriate (all *p* values are <0.001 for general group comparisons); values within a row not sharing a common superscript letter (^a,b,c,d^) are significantly different at *p* < 0.05. MUAC cut-off <24 cm for underweight [28], ≥30 cm for overweight and ≥33.5 cm for obesity [27]. Anemia is defined as hemoglobin <120 g/L for non-pregnant and <110 g/L for pregnant women and <130 g/L for men [29].

**Table 2:** Macro- and micronutrient intake of the farmers in comparison to recommended daily intake (RDI).

	All (N = 1526)	TZ-F (N = 362)	TZ-M (N = 307)	MZ-F (N = 488)	MZ-M (N = 369)	RDI/d
Energy [kcal] ^1^	2261 (1564, 2974)	2071 ^a^ (1589, 2778)	2615 ^b^ (2025, 3286)	2178 ^a^ (1373, 2912)	2211 ^a^ (1369, 3037)	1900–2800
E ≥ RDI ^2^, % (n)	46.5 (710)	51.1 (185) ^a^	48.2 (148) ^a^	51.0 (249) ^a^	34.7 (128) ^b^	
Protein [g]	60.3 (39.5, 88.9)	69.6 ^a^ (48.1, 98.8)	86.3 ^b^ (62.4, 112.5)	49.1 ^c^ (33.1, 68.7)	49.8 ^c^ (30.3, 76.2)	47–68
Pro ≥ RDI, % (n)	60.0 (915)	74.0 (268) ^a^	79.2 (243) ^a^	51.2 (250) ^b^	41.7 (154) ^c^	
Fat [g]	44.6 (30.7, 66.2)	42.0 ^a^ (26.4, 62.9)	51.0 ^b^ (35.1, 74.7)	43.5 ^a^ (32.5, 62.8)	44.7 ^a^ (28.2, 63.9)	
CHO [g]	398.3 (263.3, 528.8)	371.7 ^a^ (266.1, 479.8)	451.2 ^b^ (346.7, 553.3)	390.4 ^a^ (227.2, 523.9)	399.5 ^a^ (220.6, 543.6)	
Retinol equiv. [μg]	50.8 (24.6, 207.6)	94.4 ^a^ (24.0, 265.0)	121.5 ^a^ (29.0, 311.0)	44.2 ^b^ (24.6, 143.2)	44.2 ^b^ (23.9, 127.2)	500–800
RE ≥ RDI, % (n)	9.9 (151)	13.0 (47) ^a^	13.7 (42) ^a^	8.8 (43) ^b^	5.1 (19) ^c^	
Vitamin E [mg]	2.5 (1.2, 4.9)	1.8 ^a^ (1.5, 3.0)	3.0 ^b^ (1.5, 3.3)	2.5 ^b^ (0.9, 5.0)	2.5 ^a,b^ (0.0, 5.0)	7.5–10
VE ≥ RDI, % (n)	6.6 (101)	6.4 (23) ^a^	5.5 (17) ^a^	8.2 (40) ^a^	5.7 (21) ^a^	
Vitamin B1 [mg]	1.7 (1.1, 2.5)	1.5 ^a^ (1.0, 2.2)	1.9 ^b^ (1.4, 2.4)	1.8 ^b^ (1.1, 2.6)	1.7 ^b^ (1.0, 2.6)	1.1–1.4
B1 ≥ RDI, % (n)	73.6 (1123)	70.7 (256) ^a,c^	83.7 (257) ^b^	74.4 (363) ^a^	66.9 (247) ^c^	
Vitamin B2 [mg]	1.1 (0.7, 1.6)	1.3 ^a^ (0.8, 2.0)	1.6 ^b^ (1.1, 2.1)	0.9 ^c^ (0.5, 1.3)	0.9 ^c^ (0.5, 1.3)	1.0–1.4
B2 ≥ RDI, % (n)	46.0 (702)	61.6 (223) ^a^	64.5 (198) ^a^	39.1 (191) ^b^	24.4 (90) ^c^	
Vitamin B6 [mg]	1.9 (1.2, 2.8)	1.6 ^a^ (1.0, 2.3)	2.0 ^b^ (1.5, 2.7)	1.9 ^b^ (1.1, 3.0)	2.0 ^b^ (1.1, 3.4)	1.2–1.9
B6 ≥ RDI, % (n)	67.6 (1031)	60.8 (220) ^a^	77.2 (237) ^b^	68.4 (334) ^b,c^	65.0 (240) ^a,c^	
Folic acid [μg]	431.5 (216.5, 780.0)	611.7 ^a^ (334.5, 1011.1)	748.8 ^b^ (406.9, 1058.0)	320.0 ^c^ (177.5, 522.6)	318.7 ^c^ (161.1, 544.4)	400–600
FA ≥ RDI, % (n)	52.6 (803)	70.4 (255) ^a^	75.9 (233) ^a^	35.7 (174) ^b^	38.2 (141) ^b^	
Vitamin B12 [μg]	0 (0, 0.6)	0 ^a^ (0, 0.4)	0 ^a^ (0, 0.6)	0 ^a^ (0, 0.6)	0 ^a^ (0, 0.8)	2.4–2.6
B12 ≥ RDI, % (n)	11.8 (180)	15.2 (55) ^a^	17.9 (55) ^a^	7.2 (35) ^b^	9.5 (35) ^b^	
Vitamin C [mg]	56.5 (23.4, 1178)	36.8 ^a^ (9.8, 76.3)	39.1 ^a^ (14.0, 92.5)	77.4 ^b^ (32.9, 144.1)	68.5 ^b^ (32.1, 131.5)	40–55
VC ≥ RDI, % (n)	56.4 (860)	43.4 (157) ^a^	46.9 (144) ^a^	66.2 (323) ^b^	64.0 (236) ^b^	
Calcium [mg]	334.5 (196.0, 574.8)	357.0 ^a^ (200.8, 652.0)	403.3 ^b^ (253.0, 600.0)	280.5 ^c^ (176.6, 510.1)	312.0 ^c^ (176.9, 599.3)	1000–1300
Ca ≥ RDI, % (n)	11.2 (171)	14.6 (53) ^a^	16.0 (49) ^a^	6.8 (33) ^b^	9.8 (36) ^b^	
Magnesium [mg]	485.0 (297.4, 763.0)	420.8 ^a^ (259.6, 619.1)	465.2 ^b^ (300.0, 673.8)	552.4 ^c^ (333.5, 810.6)	553.1 ^a,c^ (293.4, 819.2)	190–260
Mg ≥ RDI, % (n)	82.6 (1261)	82.0 (297) ^a,b^	82.7 (254) ^a,b^	86.5 (422) ^a^	78.0 (288) ^b^	
Iron [mg]	20.3 (12.8, 27.9)	18.0 ^a^ (11.5, 23,5)	21.5 ^b^ (15.6, 27.6)	20.8 ^b^ (12.7, 29.3)	22.1 ^b^ (12.6, 32.7)	11–31
Fe ≥ RDI, % (n)	51.4 (784)	33.7 (122) ^a^	79.5 (244) ^b^	32.4 (158) ^a^	70.5 (260) ^c^	
Zinc [mg]	11.1 (7.0, 15.2)	11.4 ^a^ (7.2, 15.6)	13.0 ^b^ (9.5, 17.6)	10.1 ^c^ (6.4, 14.0)	10.5 ^c^ (6.1, 14.5)	9.8–19.2
Zn ≥ RDI, % (n)	44.4 (678)	56.1 (203) ^a^	43.0 (132) ^b^	49.6 (242) ^a,b^	27.4 (101) ^c^	

Data are the median (interquartile range (IQR)) ^1^ and percentage (number) ^2^. RE, retinol equivalent; RDI/day: recommended daily nutrient intake of macro- and micronutrients adjusted for age (19–50, 51–65 years), sex and pregnancy status following the FAO/WHO [35] and D-A-C-H [34] recommendations; the low bioavailability for zinc and the 10% bioavailability for iron were applied [35]. Groups were compared using the Kruskal–Wallis and pairwise comparison tests and the Chi Square test as appropriate; all *p* values are <0.001, except for EE ≥ RDI = 0.042, zinc ≥ RDI = 0.008 and Mg ≥ RDI = 0.002; villages not sharing a superscript letter (^a,b,c^) are significantly different (*p* < 0.05). RDI: energy [kcal]: female: 2300 to18 years, 2200 to 25 years, 2100 to 51 years, 2000 to 65 years, 1900 in 65 + years; trim2: +250, trim3: +500; male: 2800 to 25 years, 2700 to 51 years, 2500 to 65 years, 2500 to 65+ years; protein [g]: female: 48 to18 years; 47 to 65 years; 57 to 65+ years; pregnant: trim2: +7; trim3: +21; male: 62 to18 years, 57 to 51 years; 55 to 65 years; 67 to 65+ years; retinol equivalent s [μg]: female 19–65 years: 500; male and female 65+ years: 600; pregnant: 800; vitamin E [mg]: female: 7.5; male: 10; vitamin B1 [mg]: female: 1.1 all; pregnant: 1.4; male: 1.2 all; vitamin B2 [mg]: female: adolescents 1.0 and adults 1.1; pregnant: 1.4; male: 1.3 all; vitamin B6 [mg]: female: adolescents 1.2 and 19–50 years 1.3 and 51–65+ 1.5; pregnant: 1.9; male: adolescents–50 y: 1.3 and 51–65+ years: 1.7; FA [μg]: 400 for all; pregnant: 600; vitamin B12 [μg]: 2.4 for all; pregnant: 2.6; vitamin C [mg]: adolescents 40 and adults 45; pregnant: 55; calcium [mg]: male (19–65 years) and female (19–51 years): 1000; female adolescents, female 51+ years and male 65+ years: 1300; pregnant: 1200; magnesium [mg]: female adolescents 230; female 19–65 years and pregnant: 220; female 65+ years: 190; male: adolescents 250, male 19–65 years: 260; male 65+ years: 230; iron [mg]: female adolescents 31; female 19–51 years: 29; female 51+ years: 11; male adolescents: 19; male 19–65+ years: 14; zinc [mg]: pregnant: 14, female adolescents: 15.5, female 19–65+ years: 9.8; male: adolescents: 19.2; male 19−65+ years: 14.

**Table 3:** Frequency and amount of consumed food groups (in grams) by sex and country.

Food Intake	All (N = 1526)	TZ-F (N = 362)	TZ-M (N = 307)	MZ-F (N = 488)	MZ-M (N = 369)	*p*
Cereals, % (n) ^1^	87.6 (1337)	97.8 (354) ^a^	98.4 (302) ^a^	81.8 (399) ^b^	76.4 (282) ^c^	<0.001
Grams ^2^	625 (500, 1000)	500 (500, 1000) ^a^	1000 (500, 1000) ^b^	500 (380, 1000) ^c^	500 (500, 1000) ^d^	<0.001
Bread and prod., % (n)	15.3 (233)	25.1 (91) ^a^	28.0 (86) ^a^	5.1 (25) ^b^	8.4 (31) ^b^	<0.001
Grams	160 (100, 160)	160 (120, 160) ^a^	160 (160, 240) ^a^	100 (60, 130) ^b^	65 (60, 110) ^b^	<0.001
Starchy plants, % (n)	41.7 (637)	35.9 (130) ^a^	39.1 (120) ^a,b^	46.1 (225) ^b^	43.9 (162) ^a,b^	0.015
Grams	360 (240, 500)	300 (200, 400) ^a^	300 (200, 400) ^a,b^	400 (250, 600) ^b^	450 (250, 750) ^b^	<0.001
Legumes, % (n)	63.2 (965)	73.2 (265) ^a^	75.6 (232) ^a^	55.7 (272) ^b^	53.1 (196) ^b^	<0.001
Grams	250 (195, 500)	500 (250, 500) ^a^	500 (250, 500) ^a^	249 (125, 280) ^b^	250 (125, 280) ^b^	<0.001
DGLVs, % (n)	31.3 (477)	19.9 (72) ^a^	18.2 (56) ^a^	42.4 (207) ^b^	38.5 (142) ^b^	<0.001
Grams	250 (125, 250)	250 (125, 394) ^a^	250 (150, 500) ^a^	250 (125, 250) ^b^	185 (125, 250) ^b^	<0.001
Other vegetables, % (n)	21.5 (328)	8.6 (31) ^a^	8.8 (27) ^a^	33.4 (163) ^b^	29.0 (107) ^b^	<0.001
Grams	187.5 (125, 250)	200 (125, 250) ^a^	250 (150, 500) ^a^	145 (120, 250) ^b^	162 (120, 250) ^b^	0.282
Fruits, % (n)	9.8 (149)	19.3 (70) ^a^	17.6 (54) ^a^	2.0 (10) ^b^	4.1 (15) ^b^	<0.001
Grams	200 (120, 300)	180 (120, 255) ^a^	240 (120, 300) ^a^	200 (142, 275) ^b^	300 (200, 400) ^b^	<0.001
Nuts, % (n)	2.8 (42)	3.0 (11) ^a,b^	5.9 (18) ^a^	1.2 (6) ^b^	1.9 (7) ^b^	0.001
Grams	90 (50, 100)	100 (50, 100) ^a,b^	100 (50, 100) ^a^	40 (19, 100) ^b^	50 (25, 50) ^b^	0.081
Fish, % (n)	23.7 (362)	18.8 (68) ^a^	22.8 (70) ^a,b^	24.8 (121) ^b^	27.9 (103) ^b^	0.030
Grams	125 (90, 250)	250 (125, 250) ^a^	250 (150, 300) ^a^	100 (60, 170) ^b^	100 (70, 180) ^b^	<0.001
Meat, % (n)	12.3 (187)	7.4 (27) ^a^	11.1 (34) ^a,b^	12.5 (61) ^b, c^	17.6 (65) ^c^	<0.001
Grams	150 (100, 240)	200 (125, 300) ^a^	250 (150, 400) ^a,b^	120 (90, 180) ^a,b^	120 (90, 180) ^b^	<0.001
Eggs and diary, % (n)	2.6 (39)	4.1 (15) ^a^	4.2 (13) ^a^	1.4 (7) ^b^	1.1 (4) ^b^	0.005
Grams	120 (80, 180)	120 (80, 250) ^a^	140 (65, 340) ^a,b^	110 (80, 150) ^a,b^	70 (45, 80) ^b^	0.102
Sugar and sweets, % (n)	4.5 (69)	1.9 (7) ^a^	2.6 (8) ^a,b^	6.8 (33) ^b^	5.7 (21) ^b^	0.002
Grams	240 (87, 245)	300 (250, 400) ^a^	315 (100, 500) ^a^	240 (50, 240) ^b^	200 (75, 240) ^a,b^	0.001
Beverages, % (n)	29.8 (455)	56.9 (206) ^a^	62.9 (193) ^a^	5.9 (29) ^b^	7.3 (27) ^b^	<0.001
Grams	250 (250, 250)	250 (250, 250) ^a^	250 (250, 250) ^a^	250 (245, 250) ^b^	250 (240, 250) ^b^	<0.001

Figures are the percentages (numbers) ^1^ and the median (25th and 75th percentiles) ^2^. *p* values: the Kruskal–Wallis test and following multiple comparison tests: values within a row not sharing a common superscript letter (^a,b,c^) are significantly different at *p* < 0.05. ‘Cereals’: maize, rice, sorghum, millet (‘ugali’, ‘uji’); ‘Bread and similar products’: dough made from wheat or rice fried or baked in oil (bread, chapatti, noodles, African donuts, ‘bhajia’ (chickpea cake), and half-cake); ‘Starchy plants’: white potatoes, sweet and Irish potatoes, cassava, plantain, yam, and taro; ‘Legumes’: pigeon peas, cowpeas, kidney beans, lentils, chickpeas, and bambara nuts; ‘DGLVs’: cowpea leaves, spinach, amaranth leaves, cassava leaves, sweet potato leaves, mlenda (jute mallow), lettuce, and pumpkin leaves; ‘Other vegetables’: tomatoes, eggplant, carrots, onions, okra, cabbage, pumpkin, and mushroom; ‘Fruits’: mango, papaya, banana, jackfruit, avocado, orange, lemon, and mandarin; ‘Nuts’: groundnuts, cashews, coconut, and sesame; ‘Fish’: fresh or dried fish, sardines, fried fish, tuna, and shrimp; ‘Meat’: beef, chicken, duck, rat, goat, and pork; ‘ Eggs and diary’: egg pancakes, eggs, milk, and yoghurt; ‘Sugar and sweets’: sugar, sugarcane, carbonated drinks, cola, and sweetened juices; ‘Beverages’: tea and beer.

**Table 4:** Multiple linear regression assessing food groups consumption (in grams) as predictors of vitamin A (RE), iron and zinc intake.

Tanzania (N = 669)						Mozambique (N = 857)					
(LN) RE Intake [μg]	B	Beta	R^2^ Ch.	Partial R	%RE	(LN) RE Intake [μg]	B	Beta	R^2^ Ch.	Partial R	%RE
(Constant)	3.529366					(Constant)	3.037323				
DGLVs (g)	0.005963	0.620 **	0.328	0.625	9.1	DGLVs (g)	0.006786	0.677 **	0.363	0.654	17.0
Eggs and diary (g)	0.007574	0.220 **	0.042	0.283	1.1	Other vegetables (g)	0.002481	0.229 **	0.034	0.279	12.4
Other vegetables (g)	0.003691	0.202 **	0.033	0.259	3.4	Legumes (g)	0.001355	0.163 **	0.022	0.209	25.8
Fruits (g)	0.001419	0.102 *	0.009	0.135	6.4	Sugar and sweets (g)	0.002870	0.106 **	0.012	0.141	2.1
Starchy plants (g)	0.000854	0.103 **	0.010	0.136	18.5	Starchy plants (g)	0.000371	0.089 *	0.008	0.116	42.4
Meat (g)	0.002283	0.120 **	0.007	0.154	3.4	Eggs and diary (g)	0.008217	0.083 *	0.007	0.111	0.3
Fish (g)	0.001728	0.130 **	0.008	0.160	7.7						
Legumes (g)	0.000669	0.126 **	0.013	0.149	50.4						
(LN) **Iron Intake [mg]**	B	Beta	R^2^ Ch.	Partial R	%FE	(LN) **Iron Intake [mg]**	B	Beta	R^2^ Ch.	Partial R	%FE
(Constant)	1.816587					(Constant)	2.025				
Legumes (g)	0.001231	0.561 **	0.253	0.582	24.1	Cereals (g)	0.000807	0.507 **	0.255	0.547	53.5
Cereals (g)	0.000571	0.382 **	0.172	0.471	57.1	Starchy plants (g)	0.000636	0.245 **	0.053	0.300	20.5
DGLVs (g)	0.000933	0.233 **	0.040	0.308	4.4	Legumes (g)	0.001348	0.261 **	0.043	0.315	12.5
Starchy plants (g)	0.000516	0.150 **	0.023	0.211	8.8	Meat (g)	0.002639	0.194 **	0.027	0.239	2.0
Nuts (g)	0.003972	0.117 **	0.012	0.168	0.3	Fish (g)	0.001399	0.170 **	0.024	0.214	3.3
Fish (g)	0.000786	0.142 **	0.012	0.186	3.7	DGLVs (g)	0.000710	0.114 **	0.012	0.143	8.2
Meat (g)	0.001035	0.130 **	0.016	0.179	1.6						
(SR) **Zinc Intake [mg]**	B	Beta	R^2^ Ch.	Partial R	%ZN	(SR) **Zinc Intake [mg]**	B	Beta	R^2^ Ch.	Partial R	%ZN
(Constant)	1.731673					(Constant)	1.864001				
Legumes (g)	0.002217	0.697 **	0.318	0.751	23.2	Cereals (g)	0.001352	0.703 **	0.502	0.781	58.2
Cereals (g)	0.000762	0.353 **	0.188	0.532	54.9	Legumes (g)	0.001844	0.295 **	0.058	0.464	13.6
Fish (g)	0.003292	0.410 **	0.109	0.571	3.5	Starchy plants (g)	0.000803	0.256 **	0.052	0.415	22.3
Meat (g)	0.002692	0.234 **	0.047	0.385	1.6	Meat (g)	0.004180	0.254 **	0.059	0.412	2.2
Starchy plants (g)	0.000638	0.128 **	0.013	0.225	8.5	Fish (g)	0.001227	0.124 **	0.016	0.216	3.6
DGLVs (g)	0.000691	0.119 **	0.012	0.207	4.2	Nuts (g)	0.011565	0.078 **	0.006	0.139	0.1
Nuts (g)	0.005260	0.107 **	0.012	0.195	0.3						
Eggs and milk (g)	0.001752	0.084 **	0.008	0.153	0.5						
Bread (g)	0.000688	0.064 **	0.004	0.114	3.3						

LN = log-normal; SR = square root; B = beta coefficient; Beta = standardized beta coefficient; RE = retinol equivalents; FE = iron; ZN = zinc; * *p* < 0.05, ** *p* < 0.001 (probability of F: entry 0.01, removal 0.05), and % percentage; micronutrient contribution of consumed food group calculated by frequency and mean consumption.

## Data Availability

Not applicable.
